# Phylogenetic implications of the complete mitochondrial genome of *Ogcogastersegmentator* (Westwood, 1847) and first record of the genus *Ogcogaster* Westwood, 1847 from China (Neuroptera, Myrmeleontidae, Ascalaphinae)

**DOI:** 10.3897/BDJ.10.e85742

**Published:** 2022-07-07

**Authors:** Jingyu Wu, Yuchen Zheng, Xingyue Liu

**Affiliations:** 1 Department of Entomology, College of Plant Protection, China Agricultural University, Beijing, China Department of Entomology, College of Plant Protection, China Agricultural University Beijing China

**Keywords:** Ascalaphini, new country record, mitochondrial genome, phylogeny, Oriental Region

## Abstract

**Background:**

The genus *Ogcogaster* Westwood, 1847, which is endemic to the Oriental Region and contains only five species, has been recorded in India and Pakistan, but not in China. The genus was not sampled in any previous study on the phylogeny of Neuroptera and its affinity within Ascalaphinae is unclear.

**New information:**

The owlfly species *Ogcogastersegmentator* (Westwood, 1847) is firstly recorded from China, based on a female specimen collected from Yunnan Province, which represents the first record of the genus *Ogcogaster* from China. The complete mitochondrial genome of this species is first sequenced and described. The phylogenetic analysis, based on all 13 PCGs and two rRNA genes of the owlfly mitogenomes determined so far, assigned *O.segmentator* into a monophyletic group with *Libelloidesmacaronius* (Scopoli, 1763) and *Ascalohybrissubjacens* (Walker, 1853).

## Introduction

Owlflies, presently placed in the subfamily Ascalaphinae (Myrmeleontidae), which also includes some antlions of Palparini, Dimarini and Stilbopterygini, are a specialised lacewing group with long clavate antennae (except *Albardiafurcata* van der Weele, 1903), enlarged compound eyes and short hypostigmal cell (Machado et al. 2019), currently including three tribes, i.e. Ascalaphini, Haplogleniini and Ululodini.

The genus *Ogcogaster* Westwood, 1847, which belongs to the tribe Ascalaphini, is endemic to the Oriental Region, currently including five species (Machado et al. 2019, Oswald 2022). The adults of *Ogcogaster* are characterised by the wings with particular marking pattern: crossveins in costal area of both wings each with a dark brown marking and brownish marking posteriad pterostigma in hind-wings, the short abdomen and the long male ectoprocts extending ventrally. *Ogcogastersegmentator* (Westwood, 1847) is a remarkable species with spectacular colouration and markings as for the genus. So far, *O.segmentator* has been recorded in India and Pakistan (Ghosh and Sen 1977, Mészáros and Ábrahám 2003, Hassan and Liu 2021, Oswald 2022). The phylogenetic status of this genus in Ascalaphinae is unknown.

In this study, we first record *O.segmentator* from China and sequenced the complete mitogenome of this species. The characterisation of the *O.segmentator* mitogenome is described in detail and a phylogenetic analysis based on the mitogenome data of owlflies is presented.

## Materials and methods

### Dissection, photos and description

The classification system follows Machado et al. (2019). The terminology mainly follows Jones (2014) for body morphology, Aspöck and Aspöck (2008) for genitalia, Breitkreuz et al. (2017) for wing venation and Machado and Oswald (2020) for wing fields. Genitalia were prepared by clearing the apex of the abdomen with saturated potassium hydroxide (KOH) solution in 135℃ for seven minutes. After rinsing the KOH with distilled water, the apex of the abdomen was stained with Chlorazol Black and then transferred to glycerine for further examination. The genitalia of the specimen is preserved in microvial with glycerine and pinned beneath the specimen.

Habitus photos were taken by using Nikon^®^ D850 digital camera with AF-S Micro Nikkor 105 mm 1/2.8G ED lens. Head, thorax and female genitalia were photographed by a Nikon^®^ D850 digital camera with Laowa^®^ 25 mm F/2.8 2.5–5.0X Ultra Macro lens. Photographs were adjusted and organised with Helicon Focus v.7.7.6 (method C) and Adobe Photoshop CS 6.0.

### DNA extraction, PCR amplification and sequencing

Total genomic DNA was extracted and purified from the mid-leg muscle using TIANamp Genomic DNA Kit (Tiangen Biotech, Beijing, China). For mitochondrial genome sequencing, we constructed a 150 bp paired-end sequencing library for the specimen and used Illumina NovaSeq platform to obtain 4 Gb of sequence data. Raw data were trimmed of adapters using Trimmomatic (Bolger et al. 2014). The sample was assembled by mapping the mitogenome of *L.macaronius* (GenBank accession: FR669150) using GENEIOUS v.9.0 (Kearse et al. 2012).

### Annotation and bioinformatic analysis

Mitogenome sequence was uploaded to MITOS Web Server (http://mitos.bioinf.uni-leipzig.de/index.py) for initial mitochondrial genome annotation (Bernt et al. 2013). The tRNA genes were detected using invertebrate genetic codes. Secondary structures of the tRNAs were also predicted by MITOS. The annotation of protein coding genes (PCGs) and rRNA genes of this species was verified by hand alignment according to those of *L.macaronius* (GenBank accession: FR669150). The control region was identified by the ends of *rrnS* gene and the start of *trnI* gene. The AT and GC skews were measured using the following formulae: AT-skew = (A–T) / (A+T) and GC-skew = (G–C) / (G+C) (Perna and Kocher 1995). Nucleotide substitution rates, base composition and codon usage were analysed with MEGA 7.0 (Kumar et al. 2016). The circular map of the mitogenome was generated via CG view Server (http://cgview.ca/) (Grant and Stothard 2008).

### Phylogenetic analysis

Six species belonging to Ascalaphinae were included in our analysis for which mitochondrial genome data are available, i.e. *Ascaloptynxappendiculata* (Fabricius, 1793), *Ascalohybrissubjacens* (Walker, 1853), *Libelloidesmacaronius* (Scopoli, 1763), *Maezousumbrosus* (Esben-Petersen, 1913), *Ogcogastersegmentator* and *Suhpalacsa* sp. The outgroup taxa comprise *Bullangaflorida* (Navás, 1913), *Layahimawuzhishana* (Yang in Yang & Wang, 2002), *Chasmopterahuttii* (Westwood, 1847), *Myiodactylusosmyloides* Brauer, 1866 and *Nymphesmyrmeleonoides* Leach, 1814. GenBank accession numbers of all sequences used in this paper are listed in Table 1. The 13 PCGs alignment was conducted by Clustal W in MEGA 7.0 (Kumar et al. 2016). The rRNA genes were aligned on Gblocks v.0.91b (Castresana 2000). Phylogenetic analyses were conducted by Bayesian Inference (BI) and Maximum Likelihood (ML) methods. Bayesian analysis was performed on CIPERS Science Gateway (http://www.phylo.org) (Miller et al. 2010) with MrBayes on XEEDE (3.2.7a). Maximum Likelihood inference was conducted on IQ-tree webserver (http://iqtree.cibiv.univie.ac.at) (Nguyen, L-T et al. 2014, Trifinopoulos et al. 2016) with 1000 bootstrap replicates. The trees were visualised and edited in FigTree v.1.4.3 (Rambaut and Drummond 2016).

## Taxon treatments

### Ogcogaster

Westwood, 1847

AFD7D703-133E-5897-84FB-7C28243CFCB4

Ogcogaster Westwood, 1847 Type species: Ascalaphus (Ogcogaster) tessellatus Westwood 1847: 69. Designated by van der Weele 1908: 253.
Horischema Mészáros & Ábrahám, 2003 Type species: *Horischemaronkayorum* Mészáros and Ábrahám 2003: 341. Original designation. Synonymised by Hassan and Liu 2021: 427.
Perissoschema Mészáros & Ábrahám, 2003 Type species: *Perissoschemaevae* Mészáros and Ábrahám 2003: 346. Original designation. Synonymised by Hassan and Liu 2021: 427.
Ogcogaster Ascalaphus (Ogcogaster) segmentator Westwood, 1847

#### Diagnosis

Forewing length: 25–40 mm, hind-wing length: 21–35 mm. Antenna nearly 3/4 as long as forewing. Gena wide. Crossveins in costal area of both wings each with a dark brown marking. Brownish marking posteriad pterostigma in hind-wings. Poststigmal area generally with two rows of cells. Abdomen short, nearly half as long as hind-wing. Male ectoproct elongated, slightly curved, distally swollened with some stout setae, extending ventrally.

#### Distribution

China, Cambodia, Nepal, India, Pakistan.

### Ogcogaster segmentator

(Westwood, 1847)

07750089-A4AB-5381-851A-58DA902C2895

Ascalaphus (Ogcogaster) segmentator Westwood, 1847 Type locality: East Indies (Westwood 1847: 69).
Helicomitus salvatoris Navás, 1924 Type locality: India (Punjab, Kalka) (Navás 1924: 220). Synonymized by Sala de Castellarnau 1946: 120.
Perissoschema evae Mészáros & Ábrahám, 2003 Type locality: Pakistan (Islamabad Capital Territory, Margalla Hills, Pir Sohawa) (Mészáros and Ábrahám 2003: 346). Synonymised by Hassan and Liu 2021: 427.

#### Materials

- **Type status:** Other material. **Occurrence:** recordedBy: Yinghui Lin; individualCount: 1; sex: female; **Taxon:** class: Insecta; order: Neuroptera; family: Myrmeleontidae; **Location:** country: China; stateProvince: Yunnan; county: Dehong; verbatimLatitude: 24.8607°N; verbatimLongitude: 98.2964°E; **Event:** year: 2019; month: 09; day: 22; **Record Level:** collectionCode: CAU

#### Description

**Figure 1.**
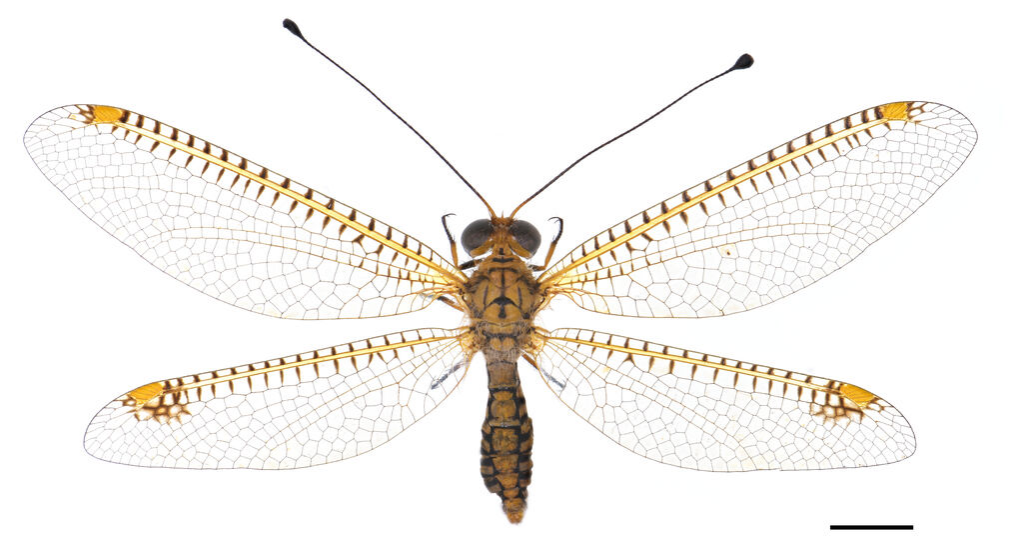
*Ogcogastersegmentator* (Westwood, 1847), female habitus. Scale bar: 10.0 mm.

**Figure 2.**
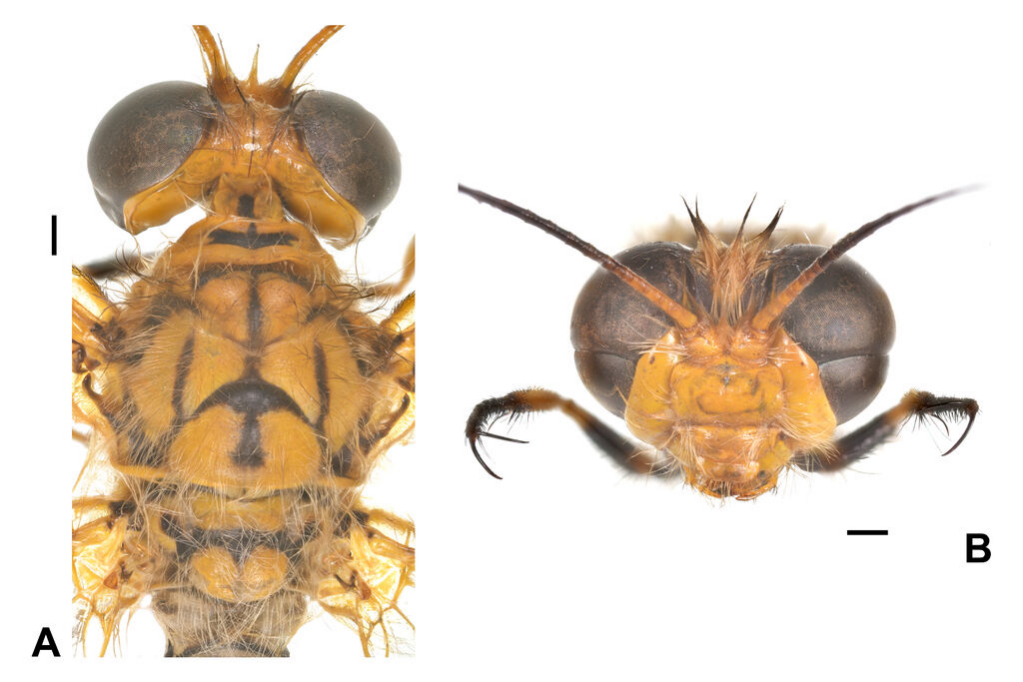
*Ogcogastersegmentator* (Westwood, 1847), female. **A** head and thorax, dorsal view; **B** head, frontal view. Scale bar: 1 mm.

**Figure 3.**
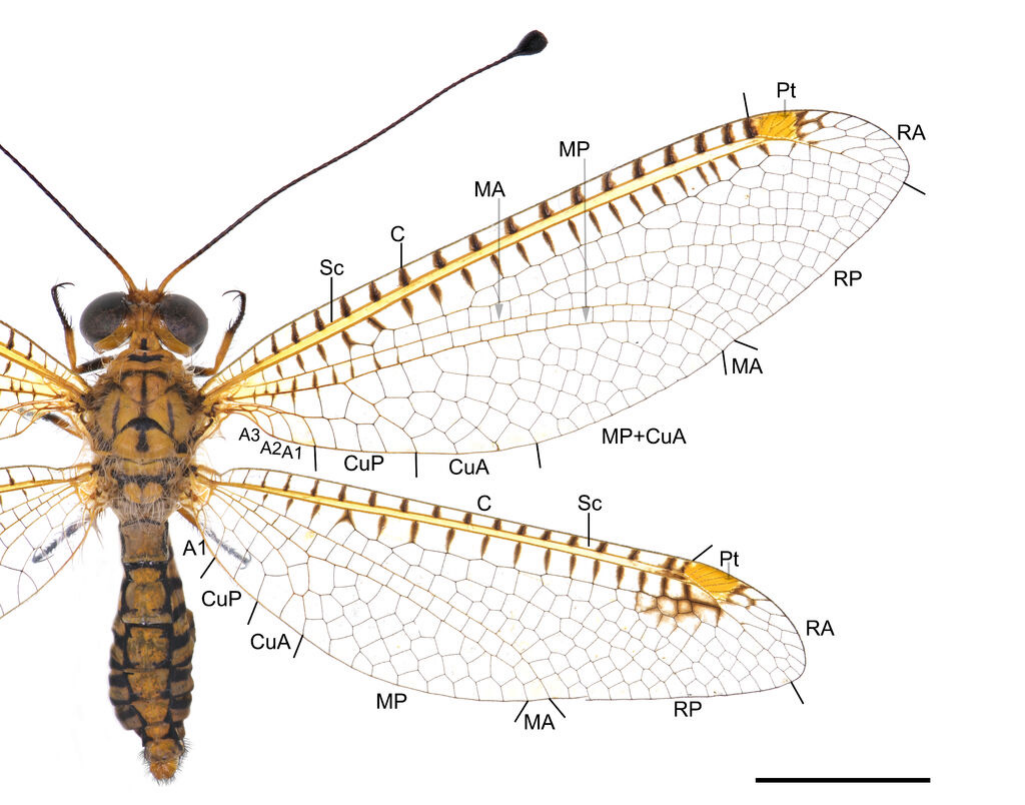
*Ogcogastersegmentator* (Westwood, 1847), female, wings. Abbreviations: C: costa; Sc: subcostal; Pt: pterostigma; RA: radius anterior; RP: radius posterior; MA: media anterior; MP: media posterior; CuA: cubitus anterior; Cup: cubitus posterior; A: anal veins. Scale bar: 10 mm.

**Figure 4.**
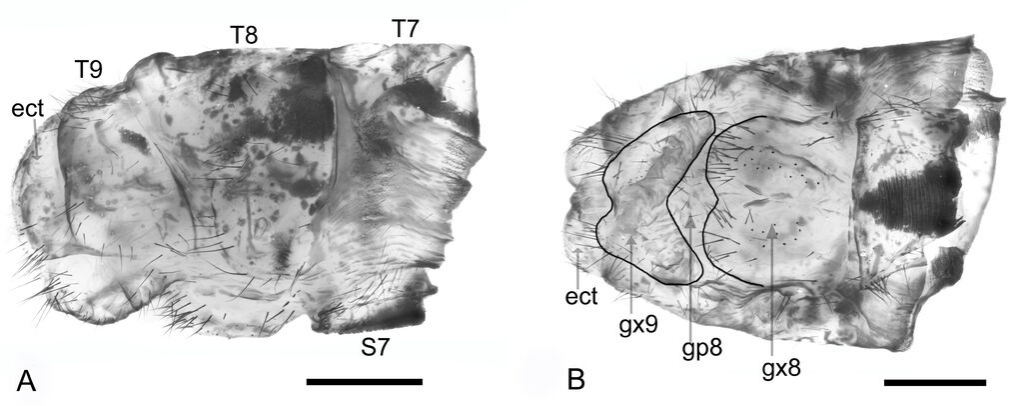
*Ogcogastersegmentator* (Westwood, 1847), female, genitalia. **A** lateral view; **B** ventral view. Abbreviation: ect: ectoproct; gx: gonocoxites; gp: gonapophyses; S: sternite; T: tergite. Scale bar: 0.5 mm.

Habitus: Fig. 1; head and thorax: Fig. 2; wing: Fig. 3; female genitalia: Fig. 4.

*Ogcogastersegmentator* (Westwood, 1847) has been described before and we re-describe it here.

Body length 27.3 mm; forewing length 30.1 mm; hind-wing length 28.5 mm.

Head. Vertex yellowish-brown, with brown setae. Scape, pedicel and basal flagellum generally yellow, remaining part of flagellum generally dark brown. Compound eye divided by a transverse furrow, with upper part larger. Frons, gena, clypeus and labrum yellow, with pale yellow setae.

Thorax. Yellow, with obvious black stripes. Pronotum medially with a transversal black marking, laterally with dense pale setae. Meso- and metathorax with dense long seate, most pale, but some brown. Mesoprescutum and mesoscutellum each with a T-shaped black marking; mesonotum laterally with a pair of discontinuous reversed U-shaped black marking. Metanotum with a black marking.

Leg. Femur yellow, medially with a black marking; tibia yellow, distally dark brown; tibial spur stout, slightly curved; tarsus dark brown; pretarsal claw slightly curved, as long as tarsomere 5. Pro- and mesotarsomere 5 as long as combined length of tarsomeres 1–4. Metatarsomere 5 shorter than combined length of tarsomeres 1–4.

Wing. Forewing subcostal area yellow; crossveins near subcostal area each with a dark brown marking; pterostigma yellow; basal mediocubital crossveins each with a dark brown marking. Five presectoral crossveins present, with a biareolate cell; RP with five branches. Hind-wing marking similar to forewing, some indistinct dark brown markings posteriad pterostigma.

Abdomen. Yellow with many black stripes. Male genitalia. Tergite 9 very large, rhomboid-shaped with yellow and brownish pattern and medium-long stiff black hairs on margin caudally. In lateral view, tergite 9 with an acute apex caudo-ventrally. Ectropocts short, stiff, black setae, stronger inside than outside; in ventral views ectoprocts short, stiff, black seate, stronger inside than outside; in ventral view ectoprocts slightly curved downwards. Gonarcus (gonocoxites 11) hood-like, hairless, fused with parameres (gonocoxites 9), anterior side of parameres with tooth. Pelta present with seate, pulvini bag-like their hairs long and brownish. Setimere black, a bit longer than hairs on pulvini (Mészáros and Ábrahám 2003). Female genitalia. Gonocoxites 8 present as a pair of ovoid structures, with sparse short setae; gonapophyses 8 small, with sparse short setae. Gonocoxites 9 base separated, apex close, with sparse short setae. Ectoproct narrowed in lateral view, distally rounded.

#### Diagnosis

Body yellow with black markings. Scape, pedicel and basal flagellum generally yellow, but remaining part of flagellum generally dark brown. Meso- and metathorax with dense long seate, most pale, but some brown. Femur medially with a black marking. Subcostal area yellow; crossveins near subcostal area each with a dark brown marking; pterostigma yellow.

#### Distribution

New record to China (Yunnan); India (Himachal Pradesh, Jammu & Kashmir, Karnataka, Maharashtra, Punjab, Uttar Pradesh); Pakistan (Azad Kashmir, Gilgit-Baltistan, Islamabad) (Ghosh and Sen 1977, Mészáros and Ábrahám 2003, Hassan and Liu 2021, Oswald 2022).

## Analysis

### Mitogenomic characterisation of Ogcogastersegmentator

**Figure 5.**
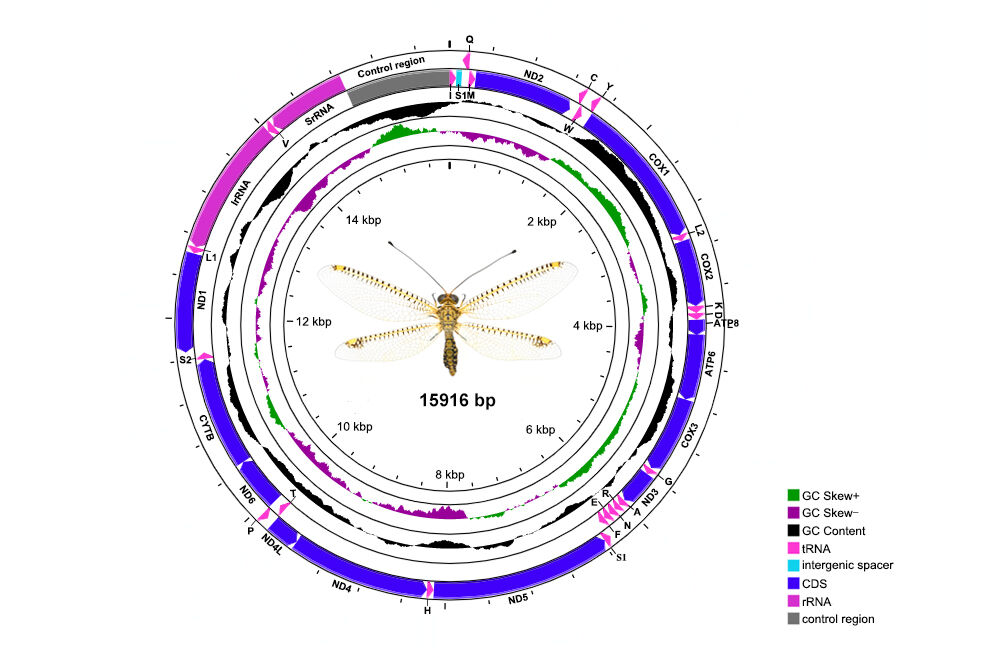
Mitochondrial map of *Ogcogastersegmentator* (Westwood, 1847).

The complete mitochondrial genome of *O.segmentator* is a typical circular, doubled-stranded molecule of 15,916 bp, with 37 genes (13 PCGs, 22 tRNAs and two rRNAs) and 15 non-coding regions. The tRNA rearrangement of *trnC*-*trnW*-*trnY* (Wang et al. 2016) occurs in the mitogenome this species. Amongst all mitochondrial genes, 14 genes (including two rRNAs, four PCGs and eight tRNAs) are situated on the light strand (L) and the rest 23 genes (including nine PCGs and 14 tRNAs) are situated on the heavy strand (H) (Fig. 5, Table 2). Four gene overlaps were detected in the mitogenome of *O.segmentator* and the length of the largest one, which is present between *atp8* and *atp6*, is only 7 bp.

The nucleotide composition of the mitogenome of *O.segmentator* is as follows: A = 40.3%, T = 34.4%, C = 14.9%, G = 10.4%, suggesting an obvious bias towards A and T. Its AT-skew and GC-skew are 0.079 and –0.178, respectively and are similar to those of *L.macaronius* (Negrisolo et al. 2011).

**Protein coding genes and codon usages.** The total length of the 13 PCGs of the *O.segmentator* mitogenome is 12,186 bp. The A+T nucleotide composition of all PCGs is 73.4%. All 13 PCGs show a positive AT-skew (0.0736) and a negative GC-skew (–0.1804). The start/stop codons of the 13 PCGs are listed in Table 2. ATN (N represents A, T, C or G) is the most commonly used start codon for PCGs in the mitogenome of *O.segmentator*. Nonetheless, the start codon of *cox1* in *O.segmentator* is ACG, which also can be found in *L.macaronius* (Negrisolo et al. 2011) and *Suhpalacsa* sp. (Song et al. 2019). The other PCGs, except *nad1*, use complete TAA or incomplete TA/T as their stop codons. Currently, the stop codon of *nad1* in most owlfly species with the mitogenomes determined is known as TAG (Beckenbach and Stewart 2009, Negrisolo et al. 2011, Cheng et al. 2013, Gao et al. 2018), but TAT for *Suhpalacsa* sp. (Song et al. 2019).

**Figure 6.**
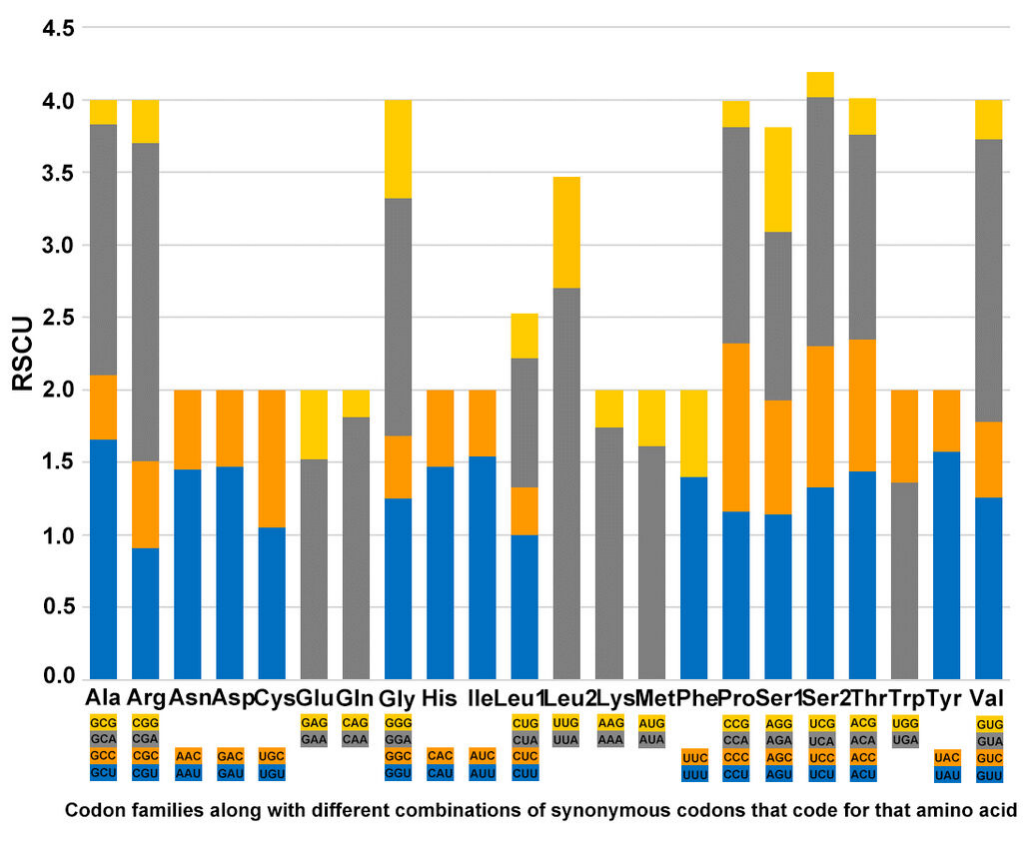
Relative synonymous codon usage (RSCU) in the mitogenome of *O.segmentator*. Codon families are provided on the x-axis along with the different combinations of synonymous codons that code for that amino acid. RSCU are provided on the y-axis.

The relative synonymous codon usage (RSCU) of the mitochondrial genes of *O.segmentator* was analysed (Fig. 6). The result suggests that the most frequently used codon is AAA-Lys (7.09%) and the least frequently used codon is CGG-Arg (0.08%). UUA-Leu2 has the highest relative synonymous codon usage (2.70), which indicates that UUA is the most preferred codon in the mitogenome of *O.segmentator*. The result also shows a preference in using A or T in the 3rd codon position of the PCGs in this species.

**Figure 7.**
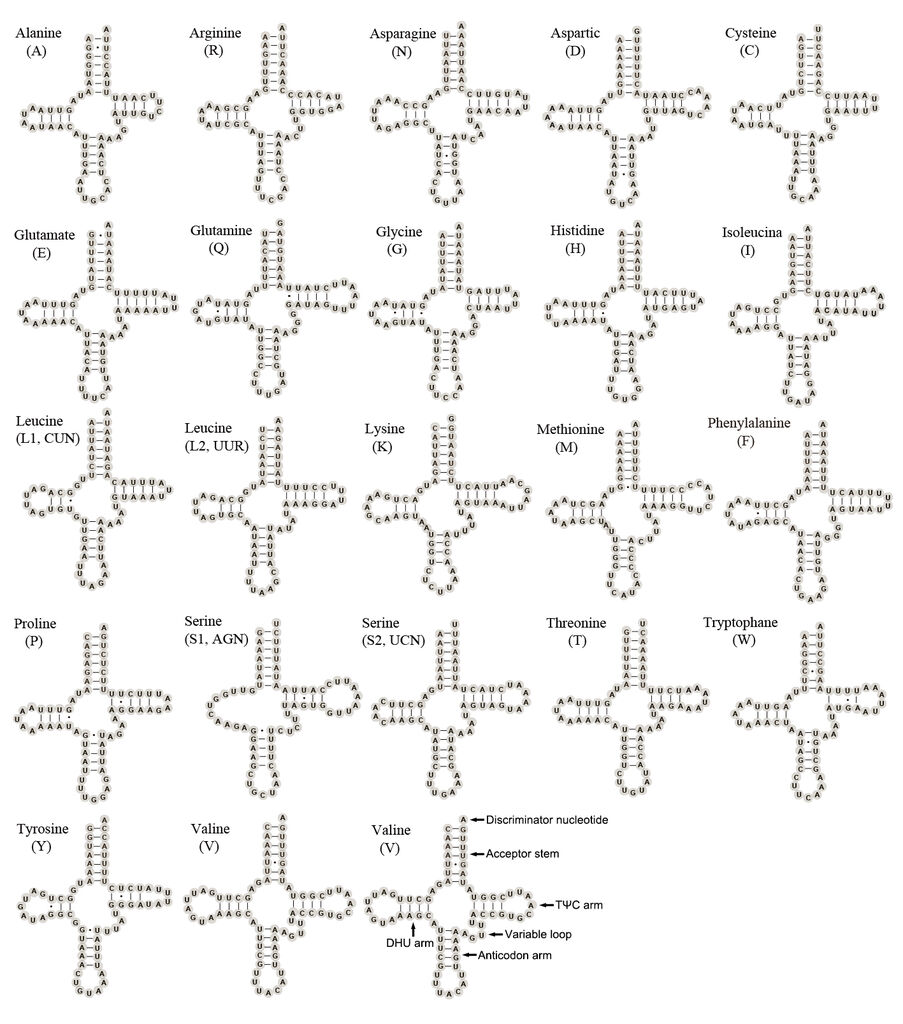
Secondary structure of the 22 tRNAs in the mitogenome of *O.segmentator*. Dash (–) indicates Waston-Crick bonds and dot (·) indicates GU bonds.

**Transfer RNA genes.** Twenty-two complete tRNA genes were detected in the mitogenome of *O.segmentator* and their secondary structures are shown in Fig. 7 respectively. The total length of the tRNAs is 1,471 bp, with length of each tRNA gene ranging from 63 bp (*trnC*) to 72 bp (*trnV*). The A+T content of the total tRNAs is 75.2%, but it shows negative AT-skew (-0.0079) and positive GC-skew (0.1566). Most tRNA genes, except *trnS1*, are folded as classical cloverleaf secondary structure. The absence of the dihydrouridine (DHU) arm in *trnS1* is a common phenomenon in insects (Wolstenholme 1992). The TΨC loop is absent in *trnC* of *O.segmentator* and in *trnF* and *trnL1* of *M.umbrosus* (Gao et al. 2018). Most base pairs conform to the classical A-U and G-C pattern matching, but there are 25 G-U mismatched base pairs in the tRNA genes of the mitogenome of *O.segmentator*. Furthermore, infrequent U-U base pairs were observed in *trnW* of *O.segmentator* and in *trnW* of *M.umbrosus* (Gao et al. 2018).

**Ribosomal RNA genes and non-coding regions.** The *rrnL* gene is located between *trnL1* and *trnV*, with the length of 1,316 bp. The *rrnS* gene is located between *trnV* and A+T-rich region, with the length of 782 bp. The total length of two rRNA genes is 2,098 bp with an average AT content of 77.1%. The AT-skew and GC-skew of all rRNAs are 0.1413 and –0.2807 respectively, suggesting the positive AT-skew and negative GC-skew of this species.

Most of non-coding regions in arthropod mitogenomes are shorter than 20 bp (Cook 2005). The mitogenome of *O.segmentator* contains 15 non-coding regions, from 1 to 1,053 bp. Two of these intervals (S1 and the A+T-rich region) are larger than 20 bp (Table 2). The intergenic spacer 1 (S1) is located between *trnI* and *trnQ*, with the length of 55 bp. This spacer is also present at the same position in the mitogenomes of other five owlfly species, from 42 to 55 bp (Beckenbach and Stewart 2009, Negrisolo et al. 2011, Cheng et al. 2013, Gao et al. 2018). The length of the A+T-rich region of *O.segmentator* is 1,053 bp and its AT and GC content are 83.8% and 16.3%, respectively. The A+T-rich region of this species shows a preference in using A over T and C over G, with the AT-skew of 0.0501 and GC-skew of –0.0184.

### Phylogeny

**Figure 8.**
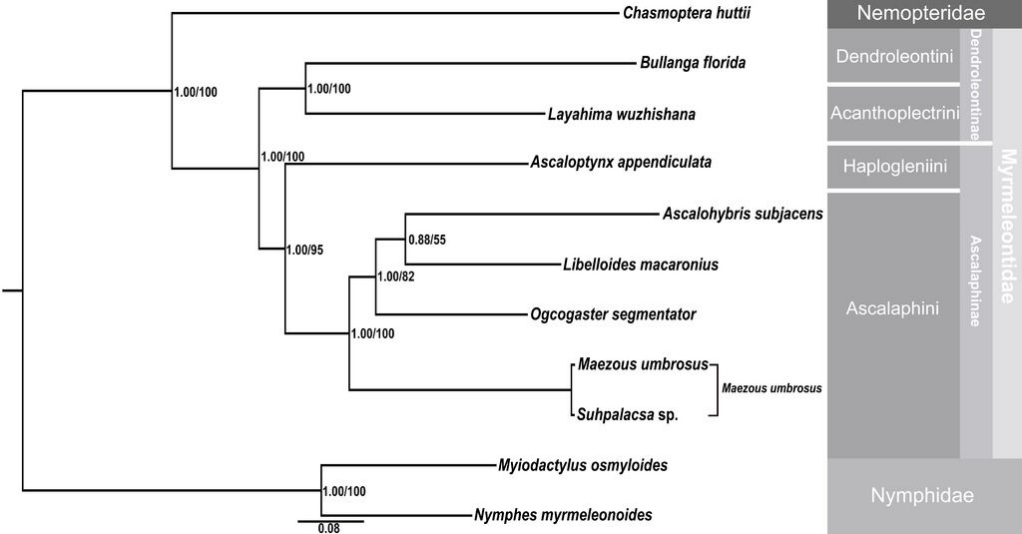
Phylogenetic tree inferred from 13 PCGs and two rRNA genes, based on BI and ML methods. Numbers at nodes are Bayesian posterior probabilities (left) and ML bootstrap values (right).

The BI and ML analyses generated trees with the same topology (Fig. 8). First, we found that *Maezousumbrosus* and *Suhpalacsa* sp. share a very long branch and have the interspecific divergence of the *cox1* between each other to be just 0.002. Therefore, the sequenced specimens probably belong to same species and, considering the synonymy between *Maezous* and *Suhpalacsa*, the two mitogenomes (GenBank accession numbers KC758703 and MK301247) should be assigned to *Maezousumbrosus* (Esben-Petersen, 1913).

All the owlflies herein sampled constitute a monophyletic clade, which is sister to the clade comprising two antlion genera of the subfamily Dendroleontinae, which, however, is a preliminary result, based on incomplete sampling. Within Ascalaphinae, *Ascalop.appendiculata* was assigned to be the sister group of the remaining owlfly species. *Ascaloh.subjacens* was recovered to be the sister group to *L.macaronius* and both species constitute a sister group of *O.segmentator*. *Maezousumbrosus* was considered to be the sister group to the lineage of *O.segmentator* + (*L.macaronius* + *Ascaloh.subjacens*).

## Discussion

The phylogeny of owlflies, especially their relationship with the antlions, has always been controversial amongst recent studies using different sets of molecular data (Wang et al. 2016, Badano et al. 2017, Michel et al. 2017, Winterton et al. 2017, Jones 2019, Machado et al. 2019, Vasilikopoulos et al. 2020). The paraphyly of Myrmeleontidae, with previous Ascalaphidae nested, was recovered in several phylogenomic studies (Wang et al. 2016, Winterton et al. 2017, Machado et al. 2019). In particular, Machado et al. (2019) proposed a new classification of Myrmeleontidae, considering Ascalaphidae as a subfamily, i.e. Ascalaphinae, which comprise six tribes, i.e. Dimarini, Palparini, Ululodini, Stilbopterygini, Haplogleniini and Ascalaphini. However, the phylogenomic analysis, based on transcriptome data (Vasilikopoulos et al. 2020) and the phylogenetic analysis, based on multi-loci data (Michel et al. 2017), recovered the monophyletic Ascalaphidae and Myrmeleontidae, respectively, although the sampling of ascalaphids in the latter study is much less than that of antlions. Based on both morphological and molecular genetic data, Badano et al. (2017) also recovered the monophyly of traditional Ascalaphidae. Moreover, Jones (2019) presented another classification different from that in Machado et al. (2019) based on the multi-loci and morphological dataset. In Jones (2019), the monophyly of Ascalaphidae was corroborated and its family rank was restored. Besides, Jones (2019) treated the aforementioned tribes Palparini and Stilbopterygini as families, i.e. Palparidae and Stilbopterygidae. Thus, the molecular phylogenetic studies, which focus on owlflies and related antlions, are still needed by greater numbers of samples.

The relationships amongst major lineages of Ascalaphinae (or previous Ascalaphidae) is also debatable. According to the traditional classification, Ascalaphidae is divided into three subfamilies (Tjeder 1992): Albardiinae, with relatively short antennae; Ascalaphinae, with the split compound eyes; and Haplogleniinae, with the non-split compound eyes. However, neither split‐eyed owlflies (Ascalaphinae or Ascalaphini) nor entire‐eyed owlflies (Haplogleniinae or Haplogleniini) were recovered to be monophyletic, either based solely on the anchored hybrid enrichment (AHE) data (Machado et al. 2019) or the mixed data with a combination of morphological characters and molecular data (Jones 2014, Jones 2019). Notably, according to Machado et al. (2019), the split-eyed Ululodini (Ululodinae in Jones 2019) is distantly related to the other split-eyed Ascalaphini (Ascalaphinae in Jones 2019), which is sister group to the entire‐eyed Haplogleniini. In addition, the entire‐eyed owlfy genus *Protidricerus* was assigned into Ascalaphinae (Ascalaphini in Machado et al. 2019) in Jones (2019). He also emphasised that the spilt compound eye that was used as a key character of owlfly taxonomy should be removed, re‐evaluated and re-interpreted.

So far, there have been very few phylogenetic studies focusing on intergeneric relationships of owlflies. The genus *Ogcogaster* was not sampled in any previous studies (Jones 2019, Machado et al. 2019). In Jones (2019), *Ascaloptynx* was assigned in a monophyletic group with several genera of Haplogleniini, but not all entire-eyed owlflies and this group was recovered to be sister group to the clade including many split-eyed genera, such as *Ascalohybris*, *Libelloides*, *Maezous* and *Suhpalacsa*. Thus, this phylogenetic framework is supported by the present result. Jones (2019) also recovered two subclades within the aforementioned clade of split-eye genera and *Ascalohybris* and *Libelloides* belong to a same subclade, while *Maezous* and *Suhpalacsa* belong to the other. Generally, our result is consistent with the result of Jones (2019). Concerning the generally concordant phylogeny recovered from a different dataset, our result suggests that the mitogenomes should be a helpful source of data for inferring the phylogeny of owlflies. In our result, *Ogcogaster* was recovered to be closer to the genera *Ascalohybris* and *Libelloides*. The similarity of morphological characteristics, especially the genital characters, amongst the three genera could also provide more support for this conclusion. A comprehensive worldwide phylogenetic analysis is needed for understanding the evolutionary history of Ascalaphinae.

## Supplementary Material

XML Treatment for Ogcogaster

XML Treatment for Ogcogaster segmentator

## Figures and Tables

**Table 1.** Taxa used in the present phylogenetic analysis.

Order	Family/Subfamily/Tribe	Species	GenBank Accession number
Neuroptera	Myrmeleontidae/Ascalaphinae/Ascalaphini	*Ogcogastersegmentator*	ON243766
	Myrmeleontidae/Ascalaphinae/Haplogleniini	*Ascaloptynxappendiculata*	FJ171324
	Myrmeleontidae/Ascalaphinae/Ascalaphini	*Ascalohybrissubjacens*	KC758703
	Myrmeleontidae/Ascalaphinae/Ascalaphini	*Libelloidesmacaronius*	FR669150
	Myrmeleontidae/Ascalaphinae/Ascalaphini	*Maezousumbrosus*	MH361300
	Myrmeleontidae/Ascalaphinae/Ascalaphini	*Suhpalacsa* sp.	MK301247
	Myrmeleontidae/Dendroleontinae/Dendroleontini	*Bullangaflorida*	KX369241
	Myrmeleontidae/Dendroleontinae/Acanthoplectrini	*Layahimawuzhishana*	MW853767
	Nemopteridae/Nemopterinae	*Chasmopterahuttii*	KT425069
	Nymphidae/Myiodactylinae	*Myiodactylusosmyloides*	KT425089
	Nymphidae/Nymphinae	*Nymphesmyrmeleonoides*	KJ461322

**Table 2.** Annotation of the complete mitogenome of *Ogcogastersegmentator* (Westwood, 1847).

Gene	Direction	Location	Size (bp)	Anticodon	Codon		Intergenic nucleotides (bp)
					Start	Stop	
trnI	J	1–68	68	GAT	-	-	55
S1	J	69–123	55	-	-	-	0
trnQ	N	124–192	69	TTG	-	-	5
trnM	J	198–266	69	CAT	-	-	0
nad2	J	267–1289	1023	-	ATT	TAA	0
trnC	N	1289–1351	63	GCA	-	-	9
trnW	J	1361–1428	68	TCA	-	-	2
trnY	N	1431–1496	66	GTA	-	-	4
cox1	J	1501–3034	1534	-	ACG	T	0
trnL2	J	3035–3099	65	TAA	-	-	2
cox2	J	3102–3783	682	-	ATG	T	0
trnK	J	3784–3854	71	CTT	-	-	1
trnD	J	3856–3923	68	GTC	-	-	0
atp8	J	3924–4082	159	-	ATT	TAA	–7
atp6	J	4076–4752	677	-	ATG	TA	0
cox3	J	4753–5541	789	-	ATG	TAA	7
trnG	J	5549–5614	66	TCC			0
nad3	J	5615–5968	354	-	ATA	TAA	6
trnA	J	5975–6039	65	TGC	-	-	3
trnR	J	6043–6105	63	TCG	-	-	3
trnN	J	6109–6176	68	GTT	-	-	2
trnS1	J	6179–6245	67	GCT	-	-	0
trnE	J	6246–6311	66	TTC	-	-	–2
trnF	N	6310–6377	68	GAA	-	-	0
nad5	N	6378–8109	1732	-	ATT	T	0
trnH	N	8110–8175	66	GTG	-	-	0
nad4	N	8176–9516	1341	-	ATG	TAA	–1
nad4L	N	9516–9803	288	-	ATG	TAA	2
trnT	J	9806–9871	66	TGT	-	-	0
trnP	N	9872–9937	66	TGG	-	-	1
nad6	J	9939–10456	518	-	ATA	TA	0
cob	J	10457–11591	1135	-	ATG	T	0
trnS2	J	11592–11658	67	TGA	-	-	17
nad1	N	11676–12629	954	-	ATA	TAG	0
trnL1	N	12630–12693	64	TAG	-	-	0
rrnL	N	12694–14009	1316	-	-	-	0
trnV	N	14010–14081	72	TAC	-	-	0
rrns	N	14082–14863	782	-	-	-	0
A+T - rich region	-	14864–15916	1053	-	-	-	0
